# Investigating the interactions of endornaviruses with each other and with other viruses in common bean, *Phaseolus vulgaris*

**DOI:** 10.1186/s12985-023-02184-y

**Published:** 2023-09-22

**Authors:** Thomas J. Brine, Satish B. Viswanathan, Alex M. Murphy, Adrienne E. Pate, Francis O. Wamonje, John P. Carr

**Affiliations:** 1 Department of Plant Sciences, University of Cambridge, Cambridge, CB2 3EA UK; 2 Pest and Pathogen Ecology, National Institute of Agricultural Botany, East Malling, ME19 6BJ UK

**Keywords:** Persistent virus, Viral mutualism, Viral synergy, Viral cross-protection

## Abstract

**Background:**

Plant viruses of the genus *Alphaendornavirus* are transmitted solely via seed and pollen and generally cause no apparent disease. It has been conjectured that certain plant endornaviruses may confer advantages on their hosts through improved performance (e.g., seed yield) or resilience to abiotic or biotic insult. We recently characterised nine common bean (*Phaseolus vulgaris* L.) varieties that harboured either Phaseolus vulgaris endornavirus (PvEV1) alone, or PvEV1 in combination with PvEV2 or PvEV1 in combination with PvEV2 and PvEV3. Here, we investigated the interactions of these endornaviruses with each other, and with three infectious pathogenic viruses: cucumber mosaic virus (CMV), bean common mosaic virus (BCMV), and bean common mosaic necrosis virus (BCMNV).

**Results:**

In lines harbouring PvEV1, PvEV1 and PvEV2, or PvEV1, PvEV2 plus PvEV3, the levels of PvEV1 and PvEV3 RNA were very similar between lines, although there were variations in PvEV2 RNA accumulation. In plants inoculated with infectious viruses, CMV, BCMV and BCMNV levels varied between lines, but this was most likely due to host genotype differences rather than to the presence or absence of endornaviruses. We tested the effects of endornaviruses on seed production and seedborne transmission of infectious pathogenic viruses but found no consistent relationship between the presence of endornaviruses and seed yield or protection from seedborne transmission of infectious pathogenic viruses.

**Conclusions:**

It was concluded that endornaviruses do not interfere with each other’s accumulation. There appears to be no direct synergy or competition between infectious pathogenic viruses and endornaviruses, however, the effects of host genotype may obscure interactions between endornaviruses and infectious viruses. There is no consistent effect of endornaviruses on seed yield or susceptibility to seedborne transmission of other viruses.

**Supplementary Information:**

The online version contains supplementary material available at 10.1186/s12985-023-02184-y.

## Background

Endornaviruses accumulate in their hosts as non-enveloped double-stranded RNA molecules, although it is now thought that they evolved from positive-sense RNA viruses [1, 2]. The monopartite RNA genomes of endornaviruses encode a single polyprotein, which contains functional protein domains corresponding to RNA-dependent RNA polymerase and RNA helicase enzymes, as well as a variety of domains that occur in some but not all endornaviruses. Plant endornaviruses are classified, along with certain others infecting fungi and oomycetes, in the genus *Alphaendornavirus* [2]. Plant endornaviruses are only transmitted vertically via seed and pollen; they are not horizontally transmissible by vectors or wounding [2]. Plant endornaviruses, as well as certain other viruses exclusively transmitted through seed and pollen (partitiviruses, for example) are sometimes referred to as ‘persistent’ viruses to distinguish them from the better understood ‘acute’ viruses, i.e., infectious viruses that cause obvious disease symptoms [3].

Endornaviruses occur in certain lineages of many wild and cultivated plant species including, among others, members of the family Fabaceae (e.g., *Vicia faba*, *Phaseolus vulgaris*), species of *Capsicum*, and cereals including rice and barley [2, 4–11]. In common bean (*P. vulgaris*), the endornaviruses Phaseolus vulgaris endornavirus (PvEV) 1, PvEV2 and PvEV3 can occur singly or in combination in various lines, but they are not present in all lineages [4, 8, 11, 12].

It has been proposed that endornaviruses may provide benefits to their hosts [13], giving an example of virus-host mutualism [14]. This may perhaps explain why these inherited viruses are so widespread and persist over many generations. The demonstration that common bean plants of the Black Turtle Soup type carrying PvEV1 and PvEV2 yielded longer pods and produced a greater mass of seeds than non-carriers [15], supports the idea that plant endornaviruses may be mutualistic. However, in broad bean (*V. faba*), a double-stranded RNA (later confirmed to be an endornavirus) conferred a male sterility phenotype [16], which argues against a beneficial role. A recent survey of common bean varieties, that are popular in East Africa, identified lines of plants containing PvEV1, PvEV1 and PvEV2, and PvEV1, PvEV2 plus PvEV3 [4]. However, it is not known if or how these endornaviruses modify the phenotypes of plants of these common bean lines.

Common bean is an important crop in East and Central Africa, where it is an essential source of dietary protein and carbohydrates. Common bean is also rich in iron and zinc, which are of vital importance in this region, which has a high incidence of anaemia [17–19]. Mixed cropping systems in East and Central Africa often include common bean or other legumes as intercrops. This is because it enriches the soil with fixed nitrogen to support cultivation of other crops including, among others, maize, banana, and potato [20]. Acute viruses, including the potyviruses bean common mosaic virus (BCMV) and bean common mosaic necrosis virus (BCMNV) as well as cucumber mosaic virus (CMV), a cucumovirus, cause a range of disease symptoms including stunting, developmental abnormalities, and can decreases in crop yield [21–24]. In contrast to the endornaviruses, which induce no obvious disease symptoms and that are not horizontally transmissible, BCMV, BCMNV and CMV are efficiently transmitted horizontally by aphid vectors and wounding, as well as vertically through seed [21, 24, 25]. In previous work we characterised endornaviruses present in a range of common bean lines that are grown as crops in east Africa [4]. In this work we investigated how these persistent viruses interact with each other, with acute viruses, if endornaviruses affect seed number and weight, and if seedborne transmission of BCMV or CMV is affected by endornaviruses.

## Methods

### Plant materials and growth conditions

The common bean varieties used in this study were described in a previous paper and sourced through the bean research programmes of the Kenya Agricultural and Livestock Research Organisation (KALRO) in Kenya and the International Centre for Tropical Agriculture (CIAT) in Uganda [4]. A list of lines not carrying endornaviruses and lines that do harbour endornaviruses, together with their complements of PvEV1, PvEV2 or PvEV3, is provided in Additional file 1: Table S1. Seeds were germinated as previously described and seedlings transplanted to pots filled with a 6:1 mixture of Levington M3 compost (ICL Professional Agriculture, Ipswich, UK) and horticultural shard sand (Melcourt, Tetbury, UK). Plants for most experiments were grown in a Conviron (Winnipeg, Manitoba, Canada) controlled environment room at 22 °C, 60% humidity, under illumination with 200 μE m^−2^ s^−1^ photosynthetically active radiation for 16 h per day. Plants used for measurement of the rate of viral seedborne transmission were grown in a glasshouse maintained at approximately 18 °C during the day and 15 °C at night, with supplementary lights activated between 04.00 and 20.00 when ambient light levels dropped below 150 W/m^2^.

### Inoculation of common bean plants with CMV, BCMV and BCMNV and measurement of seed production

The infectious viruses bean common mosaic virus (potyvirus, BCMV) isolate PV-0915, bean common mosaic necrosis virus (potyvirus, BCMNV) isolate PV-0413, and cucumber mosaic virus (cucumovirus, CMV) isolate PV-0473 were obtained from the German Collection of Microorganisms and Cell Cultures (DSMZ: https://www.dsmz.de/collection/catalogue). Mechanical inoculation using infected plant sap (or mock inoculation with sterile water) onto the first two true leaves of bean plants has been described by Wamonje and colleagues [26]. Systemic infection of plants with CMV, BCMV and BCMNV was authenticated by double-antibody sandwich enzyme-linked immunosorbent assays (DAS-ELISA), using sera corresponding to the coat proteins of each of the viruses (Bio-Reba, Reinach, Switzerland).

Seeds were collected from plants between two to three months post inoculation when seed pods had fully dried out, and they were weighed (20 seeds per cultivar per treatment) using a Mettler Toledo AX105 Analytical SemiMicro balance (Columbus, OH, USA). Seeds were germinated and the first true leaves of the seedlings were sampled and used for DAS-ELISA to detect CMV and BCMV infections. Measurements of seed mass and numbers were analysed using R v.4.2.1in Rstudio (Rstudio, PBC, Boston, MA, USA) [27], using one-way ANOVA and unpaired samples t-tests. Pairwise comparisons following a significant test result from one-way ANOVA were performed using Tukey’s Honest Significant Difference post hoc test. Data for seedborne transmission for infectious viruses was analysed using binomial regression models in Rstudio, with *p-*values < 0.05 considered to be statistically significant.

### Determination of viral RNA steady-state levels by reverse transcription coupled quantitative polymerase chain reaction (RT-qPCR) assays

RNA extractions were performed on samples of approximately 50 mg of trifoliate leaf tissue using the NORGEN Total RNA Purification Plus Kit (NORGEN Biotek, Thorold, Ontario, Canada). The concentrations and purity of RNA extracts were determined spectrophotometrically using a NanoDrop 2000 spectrophotometer (Thermo Scientific, Waltham, MA, USA). Total plant RNA was heated to 90 ºC and rapidly cooled on ice before being reverse transcribed using GoScript (Promega) with random primers. The cDNA samples were used for quantitative polymerase chain reaction (qPCR) assays using the BioLine SensiMix SYBR No-ROX kit in a CFX Connect Real-Time PCR System (Bio-Rad, CA, USA) using appropriate primers (Additional file 1: Table S2). The Ct values for the three technical replicates were averaged to give a mean Ct value. Mean relative PvEV RNA steady-state accumulation was calculated from these mean Ct values using the Pfaffl method [28]. PvEV RNA accumulation data was calculated relative to transcripts of two common bean ‘housekeeping’ genes, *PvActin 11* and *PvUnknown 1* (NCBI GI 187435357), using primers designed specifically for RT-qPCR [29]. These were identified by Borges et al. [29] as suitably stable control transcripts in common bean plants exposed to biotic and abiotic stresses. Primers used for amplification of viral RNA sequences and host housekeeping transcripts are listed in Additional file 1: Table S2. Experiments were carried out at least three times.

## Results

### Endornavirus RNA accumulation varies between host backgrounds

We examined the relative accumulation of endornavirus RNAs in nine cultivars of common bean containing PvEV1. Single PvEV1 infections are known to be present in lines RED40, RWR1668 and GLP1127; lines KK022, SER16 and RWR2245 contain both PvEV1 and PvEV2, and PvEV1, PvEV2 and PvEV3 are present in the lines KK072, RWR2075 and MCM2001 (Additional file 1: Table S1) [4]. The steady state accumulation of endornavirus RNAs was measured, relative to accumulation of two housekeeping gene transcripts, *PvActin 11* and *PvUnknown 1*, using RT-qPCR assays (Additional file 1: Table S2), the expression of which was stable across treatments (see raw RT-qPCR data presented in Additional file 2: Spreadsheet S1).

The accumulation of PvEV1 RNA did not vary significantly between the nine varieties, which harboured this endornavirus (Kruskal–Wallis test, χ^2^ = 9.2722, df = 8, *p =* 0.3199, n = 90), including in lines that also harboured PvEV2 (KK022, SER16 and RWR2245) or PvEV2 plus PvEV3 (KK072, RWR2075 and MCM2001) (Table 1). Six cultivars contained PvEV2, together with PvEV1 (KK022, SER16 and RWR2245) or with PvEV1 and PvEV3 also present (KK072, RWR2075 and MCM2001). There were differences in accumulation of PvEV2 RNA accumulation across the six cultivars analysed (χ^2^ = 45.511, df = 5, *p =* 1.142e-08, n = 60) (Table 1). The pattern of relatively higher or lower steady state accumulation for PvEV2 RNA did not appear to reflect any clear relationship to the presence of either PvEV1 or PvEV3. In the PvEV1 containing cultivars, PvEV2 RNA accumulation was similar in cultivars KK022 and RWR2245 but significantly lower in line SER16. In the lines harbouring all three of the endornaviruses, PvEV2 RNA accumulation was similar in lines KK072 and RWR2075 (although lower than in lines KK022 and RWR2245) and markedly lower in the line MCM2001 (Table 1). In plants of lines KK072, RWR2075 and MCM2001 there were no significant differences in PvEV3 RNA accumulation (Table 1).

**Table 1:** The relative steady-state RNA accumulation for the endornaviruses PvEV1, PvEV2 and PvEV3 in nine *P. vulgaris* cultivars

Plant Line	Mean relative endornavirus RNA accumulation ± SEM*		
	PvEV1	PvEV2	PvEV3
RED40	4.28 ± 0.96	n/a	n/a
RWR1668	5.01 ± 1.31	n/a	n/a
GLP1127	2.73 ± 0.96	n/a	n/a
KK022	4.13 ± 1.34	0.40 ± 0.15^a^	n/a
SER16	7.38 ± 2.78	0.0052 ± 0.0035^b^	n/a
RWR2245	4.54 ± 1.57	0.32 ± 0.12^a^	n/a
KK072	2.43 ± 1.26	0.049 ± 0.016^c^	0.028 ± 0.0086
RWR2075	7.08 ± 2.29	0.064 ± 0.016^c^	0.024 ± 0.0062
MCM2001	8.97 ± 2.39	0.0018 ± 0.00054^b^	0.013 ± 0.0026

^*^ Reverse transcription-quantitative polymerase chain reaction assays with appropriate primers (Additional file 1: Table S2) were used to measure endornavirus RNA accumulation in nine cultivars of *Phaseolus vulgaris* (Additional file 1: Table S1), relative to accumulation of two housekeeping gene transcripts, *PvActin 11* and *PvUnknown 1* [29]. There were no statistically significant differences (*p* < 0.05) in relative RNA steady-state accumulation across the cultivars for PvEV1 as determined by Kruskal–Wallis (χ^2^ = 9.2722, df = 8, *p =* 0.3199, n = 90), or for PvEV3 (χ^2^ = 2.0004, df = 2, *p =* 0.3678, n = 30). Statistically significant differences (p < 0.05) in PvEV2 RNA accumulation as determined by Kruskal–Wallis (χ^2^ = 45.511, df = 5, *p =* 1.142e-08, n = 60) are indicated by different lower-case letters. Pairwise comparisons were performed using a Wilcoxon rank sum test with Benjamini–Hochberg *p-*value correction. RNA samples were isolated from 10 individual plants of each line to assay for each endornavirus (i.e., n = 10 plants per line per endornavirus). ‘n/a’ indicates not assayed, i.e., plant line does not contain the indicated endornavirus

### Acute virus infection can alter the accumulation of endornavirus RNAs

We investigated if accumulation of the RNAs of PvEV1, PvEV2 or PvEV3 is increased or decreased in leaf tissue systemically infected with CMV, BCMV or BCMNV. Systemic infection with these three acute viruses was confirmed using DAS-ELISA. Most of this work was carried out using CMV, since the lines RWR1668, GLP1127, KK022, SER16, KK072, RWR2075 and MCM2001 carry immunity to BCMV and BCMNV conferred by the recessive resistance gene *bc-3* in combination with the dominant *I* gene [30, 31].

The effects of CMV infection on accumulation of PvEV1, 2 and 3 RNA varied between lines. In plants of five lines (RED40, RWR1668, GLP1127, KK072 and RWR2075) CMV did not induce changes in PvEV1 RNA accumulation (W = 30, *p-*value = 0.1321, n = 21; W = 66, *p-*value = 0.2475, n = 20; W = 66, *p-*value = 0.4679, n = 21; W = 23, *p-*value = 0.2698, n = 17; W = 30, *p-*value = 0.1431, n = 20; respectively), whilst CMV induced decreases in PvEV1 RNA levels in KK022 (W = 89, *p-*value = 0.001643, n = 20), SER16 (W = 58, *p-*value = 0.02499, n = 17) and MCM2001 (W = 94, *p-*value = 0.0003248, n = 20), and an increase in PvEV1 RNA levels in RWR2245 (W = 20, *p-*value = 0.04347, n = 19) (Table 2). The effect of CMV infection on PvEV2 RNA accumulation was analysed in six cultivars containing PvEV2. In plants of SER16, RWR2245, KK072 and RWR2075 there were no significant changes in PvEV2 RNA accumulation following CMV infection (W = 43, *p-*value = 0.4747, n = 17; W = 38, *p-*value = 0.6038, n = 19; W = 20, *p-*value = 0.1613, n = 17; W = 44, *p-*value = 0.6842, n = 20; respectively). However, PvEV2 RNA accumulation was diminished in CMV-infected KK022 (W = 89, *p-*value = 0.001643, n = 20) and MCM2001 (W = 100, *p-*value = 1.083 × 10^–5^, n = 20) plants (Table 2). The effect of CMV on PvEV3 RNA accumulation was analysed in three lines, of which two (KK072 and RWR2075) showed no changes in PvEV3 RNA accumulation (W = 17, *p-*value = 0.08782, n = 17; W = 56, *p-*value = 0.6842, n = 20; respectively), whilst plants of MCM2001 exhibited a decline in PvEV3 RNA accumulation (W = 100, *p-*value = 1.083 × 10^–5^, n = 20) (Table 2). Only in plants of line MCM2001 was CMV seen to induce decreases in the accumulation of all three endornaviruses.

**Table 2:** The effects of cucumber mosaic virus infection on the accumulation of endornavirus RNAs

Plant Line	Mean relative endornavirus RNA accumulation ± SEM*					
	PvEV1		PvEV2		PvEV3	
	Mock	CMV	Mock	CMV	Mock	CMV
RED40	1.20 ± 0.23	4.51 ± 1.39	n/a	n/a	n/a	n/a
RWR1668	1.31 ± 0.30	0.88 ± 0.24	n/a	n/a	n/a	n/a
GLP1127	1.37 ± 0.28	1.33 ± 0.51	n/a	n/a	n/a	n/a
KK022	1.91 ± 0.89^a^	0.25 ± 0.056^b^	1.77 ± 0.67^a^	0.32 ± 0.18^b^	n/a	n/a
SER16	1.11 ± 0.20^a^	0.43 ± 0.20^b^	1.93 ± 0.88	4.17 ± 2.67	n/a	n/a
RWR2245	1.22 ± 0.26^a^	5.98 ± 2.97^b^	1.15 ± 0.20	7.31 ± 3.43	n/a	n/a
KK072	1.50 ± 0.55	5.28 ± 1.50	1.18 ± 0.29	9.48 ± 3.86	1.56 ± 0.59	6.37 ± 1.73
RWR2075	1.76 ± 0.61	2.70 ± 0.53	1.11 ± 0.17	1.16 ± 0.16	1.06 ± 0.13	1.33 ± 0.33
MCM2001	1.65 ± 0.52^a^	0.11 ± 0.026^b^	1.17 ± 0.22^a^	0.11 ± 0.012^b^	2.48 ± 0.58^a^	0.28 ± 0.062^b^

^*^Reverse transcription-quantitative polymerase chain reaction assays with appropriate primers (Table S2) were used to measure endornavirus RNA accumulation in nine cultivars of *Phaseolus vulgaris* (Table S1), relative to accumulation of two housekeeping gene transcripts, *PvActin 11* and *PvUnknown 1* [29]. Plants were either infected with cucumber mosaic virus (CMV) or mock inoculated. Statistically significant differences (*p* < 0.05) in PvEV1, 2 and 3 RNA accumulation between mock-inoculated and infected with CMV, as determined by Wilcoxon Rank sum test, are indicated by different lower-case letters. RNA samples were isolated from 10 individual plants of each line to assay for each endornavirus (i.e., n = 10 plants per line per endornavirus) with the following exceptions: RED40 CMV infected (n = 11); GLP1127 mock-inoculated (n = 11); KK022 mock-inoculated (n = 9); KK022 CMV infected (n = 11); SER16 mock-inoculated (n = 7); RWR2245 mock-inoculated (n = 9); KK072 mock-inoculated (n = 7). ‘n/a’ indicates not assayed, i.e., plant line does not contain the indicated endornavirus

The effects of BCMV and BCMNV on PvEV1 RNA accumulation were analysed in plants of the RED40 line (which harbours only one endornavirus, PvEV1) and of line RWR2245, which contains PvEV1 and PvEV2. In RED40 plants, neither BCMV nor BCMNV affected PvEV1 RNA accumulation (W = 42, *p-*value = 0.5787, n = 20; W = 53, *p-*value = 0.8534, n = 20; respectively) (Tables 3; 4), while in RWR2245 plants BCMV induced a significant increase in PvEV1 RNA accumulation (W = 9, *p-*value = 0.0021, n = 19) (Table 3), but BCMNV infection resulted in a significant decrease (W = 90, *p-*value = 2.165 × 10^–5^, n = 19) (Table 4). With this collection of lines, it was only possible to analyse the effects of BCMV and BCMNV on accumulation of PvEV2 RNA in the line RWR2245. Interestingly, these two, closely related potyviruses induced contrasting effects, with BCMV inducing an increase in PvEV2 RNA accumulation (W = 9, *p-*value = 0.0021, n = 19) (Table 3), and BCMNV inducing a significant decrease in the level of PvEV2 RNA (W = 90, *p-*value = 2.165 × 10^–5^, n = 19) (Table 4).

**Table 3:** The effects of bean common mosaic virus infection on the accumulation of endornavirus RNAs

Plant Line	Mean relative endornavirus RNA accumulation ± SEM*			
	PvEV1		PvEV2	
	Mock	BCMV	Mock	BCMV
RED40	1.20 ± 0.23	1.98 ± 0.61	n/a	n/a
RWR2245	1.22 ± 0.26^a^	5.52 ± 1.56^b^	1.15 ± 0.20^a^	3.08 ± 0.81^b^

^*^Reverse transcription-quantitative polymerase chain reaction assays with appropriate primers (Additional file 1:Table S2) were used to measure endornavirus RNA accumulation in two cultivars of *Phaseolus vulgaris* (Additional file 1:Table S1), relative to accumulation of two housekeeping gene transcripts, *PvActin 11* and *PvUnknown 1* [29]. Plants were either infected with bean common mosaic virus (BCMV) or mock inoculated. Statistically significant differences (*p* < 0.05) in PvEV1 and 2 RNA accumulation between mock-inoculated and infected with BCMV, as determined by Wilcoxon Rank sum test, are indicated by different lower-case letters. RNA samples were isolated from 10 individual plants of each line to assay for each endornavirus (i.e., n = 10 plants per line per endornavirus) with the following exception: RWR2245 mock-inoculated (n = 9)

**Table 4:** The effects of bean common mosaic necrosis virus infection on the accumulation of endornavirus RNAs

Plant Line	Mean relative endornavirus RNA accumulation ± SEM*			
	PvEV1		PvEV2	
	Mock	BCMNV	Mock	BCMNV
RED40	1.20 ± 0.23	1.64 ± 0.64	n/a	n/a
RWR2245	1.22 ± 0.26^a^	0.018 ± 0.0048^b^	1.15 ± 0.20^a^	0.017 ± 0.0031^b^

^*^Reverse transcription-quantitative polymerase chain reaction assays with appropriate primers (Additional file 1:Table S2) were used to measure endornavirus RNA accumulation in two cultivars of *Phaseolus vulgaris* (Additional file 1:Table S1), relative to accumulation of two housekeeping gene transcripts, *PvActin 11* and *PvUnknown 1* [29]. Plants were either infected with bean common mosaic necrosis virus (BCMNV) or mock inoculated. Statistically significant differences (*p* < 0.05) in PvEV1 and 2 RNA accumulation between mock-inoculated and infected with BCMNV, as determined by Wilcoxon Rank sum test, are indicated by different lower-case letters. RNA samples were isolated from 10 individual plants of each line to assay for each endornavirus (i.e., n = 10 plants per line per endornavirus) with the following exception: RWR2245 mock-inoculated (n = 9)

### Accumulation of acute virus RNA is affected more by host background than by the presence of endornaviruses

Fourteen days following mock-inoculation or inoculation of plants on lower leaves, RNA was extracted from upper, non-inoculated leaves and used for RT-qPCR for CMV, BCMV, or BCMNV. Viral RNA accumulation was normalized to the accumulation of the host housekeeping transcripts *PvActin 11* and *PvUnknown 1* [29]. CMV RNA accumulation in systemically infected leaves varied by up to an order of magnitude between plants of the nine common bean lines (Table 5). There were statistically significant differences (p < 0.05) in relative CMV RNA steady-state accumulation across the cultivars as determined by the Kruskal–Wallis test (χ^2^ = 33.018, df = 11, *p =* 0.0005226, n = 120). However, when plant lines were grouped 1 to 3 in increasing order of CMV RNA accumulation (Table 5), it was found that despite an overall significant Kruskal–Wallis test result, pairwise comparisons performed using a Wilcoxon rank sum test with Benjamini–Hochberg *p-*value correction did not reveal any significant differences in relative CMV RNA accumulation between groups. The *p-*values from the post hoc Wilcoxon rank sum test are presented in Table 6 and, in some instances, there were statistically significant pairwise between-line differences in CMV RNA accumulation. These between-line variations in CMV RNA accumulation, including those that were statistically significant, did not relate to the absence or presence of the endornaviruses, since the three endornavirus-free lines (GLP24, KATX56 and Wairimu Dwarf) also varied in CMV accumulation (Tables 5, 6).

**Table 5:** The effects of *Phaseolus vulgaris endornavirus* (PvEV) 1, PvEV2 and PvEV3 on the accumulation of cucumber mosaic virus RNA accumulation

Endornavirus Content	Plant Line	Mean relative CMV RNA accumulation ± SEM*	Group
No endornavirus	GLP24	9.95 ± 5.11	2
	KATX56	3.19 ± 1.37	1
	Wairimu	21.48 ± 13.19	3
PvEV1	RED40	10.48 ± 3.03	2
	RWR1668	32.42 ± 9.90	3
	GLP1127	21.67 ± 7.88	3
PvEV1 & PvEV2	KK022	3.60 ± 2.75	1
	SER16	2.23 ± 0.89	1
	RWR2245	30.50 ± 10.72	3
PvEV1, PvEV2 & PvEV3	KK072	8.63 ± 4.52	2
	RWR2075	17.16 ± 7.93	3
	MCM2001	3.18 ± 2.60	1

*Reverse transcription-quantitative polymerase chain reaction assays with appropriate primers (Table S2) were used to measure endornavirus RNA accumulation in twelve cultivars of *Phaseolus vulgaris* (Table S1), relative to accumulation of two housekeeping gene transcripts, *PvActin 11* and *PvUnknown 1* [29]. Plants were infected with cucumber mosaic virus (CMV). Plant lines were assigned to groups of low (1), medium (2) or high (3) CMV RNA accumulation based on inspection of mean values and *p-*values but were not statistically significantly different at a 95% confidence interval. RNA was isolated from 10 individual plants of each line to assay for each endornavirus (i.e., n = 10 plants per line per endornavirus) with the following exceptions: KATX56 and KK022 (n = 11). Wairimu = Wairimu Dwarf

**Table 6:** Summary of pairwise comparisons of relative steady-state accumulation of cucumber mosaic virus RNA in common bean lines showing *p-*values

Endornavirus content		Endornavirus-free			PvEV1			PvEV1 & PvEV2			PvEV1, PvEV2 & PvEV3	
	Plant Line	GLP24	KATX56	Wairimu	RED40	RWR1668	GLP1127	KK022	SER16	RWR2245	KK072	RWR2075
Endornavirus-free	KATX56	0.564										
	Wairimu	0.671	0.281									
PvEV1	RED40	0.540	0.142	0.818								
	RWR1668	0.183	**0.038**	0.286	0.218							
	GLP1127	0.284	0.070	0.506	0.682	0.623						
PvEV1 & PvEV2	KK022	0.506	0.682	0.218	0.089	**0.037**	**0.048**					
	SER16	0.940	0.700	0.419	0.089	**0.038**	**0.045**	0.506				
	RWR2245	0.207	**0.037**	0.506	0.419	0.985	0.717	**0.037**	0.069			
PvEV1, PvEV2 & PvEV3	KK072	1.000	0.531	0.564	0.506	0.183	0.218	0.281	0.682	0.183		
	RWR2075	0.369	0.124	0.847	0.940	0.399	0.682	0.081	0.058	0.506	0.506	
	MCM2001	0.419	0.818	0.286	0.058	**0.037**	**0.037**	0.818	0.280	**0.037**	0.399	**0.037**

The *p-*values generated from multiple Wilcoxon rank sum tests with Benjamini–Hochberg *p-*value corrections. Data highlighted bold denote statistically significant differences in relative steady-state accumulation of cucumber mosaic virus RNA between common bean lines

Measurements of BCMV RNA accumulation carried out in plants of three lines of common bean, none of which contain endornaviruses (GLP24, KATX56, and Wairimu Dwarf), showed statistically significant differences between mean values for all three, with the mean value being an order of magnitude greater in Wairimu Dwarf, than in GLP24 (Table 7). The levels of BCMV accumulation in plants of lines RED40 (which contains PvEV1) and RWR2245 (which contains PvEV1 and PvEV2) were not significantly different to levels in Wairimu Dwarf (Table 7). Using the same plant lines, BCMNV RNA accumulated to similar levels in GLP24, KATX56 and Wairimu Dwarf (which contain no endornaviruses), as well as in RED40 (which contains PvEV1), with KATX56 showing the lowest mean accumulation that was lower to a statistically significant extent than in GLP24 and RED40 (Table 8). Notably, the accumulation of BCMNV RNA in systemically infected leaves of RWR2245 plants was two orders of magnitude lower than in comparable tissues of plants of the other four lines tested (Table 8).

**Table 7:** The effects of *Phaseolus vulgaris Endornaviruses* 1 and 2 on the accumulation of bean common mosaic virus RNA accumulation

Endornavirus Content	Plant Line	Mean relative BCMV RNA accumulation ± SEM*
Endornavirus-free	GLP24	0.41 ± 0.071^a^
	KATX56	0.77 ± 0.11^b^
	Wairimu	6.03 ± 1.44^c^
PvEV1	RED40	4.42 ± 1.55^bc^
PvEV1 & PvEV2	RWR2245	7.29 ± 2.22^c^

^*^Reverse transcription-quantitative polymerase chain reaction assays with appropriate primers (Table S2) were used to measure endornavirus RNA accumulation in twelve cultivars of *Phaseolus vulgaris* (Table S1), relative to accumulation of two housekeeping gene transcripts, *PvActin 11* and *PvUnknown 1* [29] in plants of indicated lines infected with BCMV. There were statistically significant differences (*p* < 0.05) in relative BCMV RNA steady-state accumulation across the cultivars as determined by Kruskal–Wallis (χ^2^ = 28.334, df = 4, *p =* 0.00001067, n = 50). Pairwise comparisons were performed using a Wilcoxon rank sum test with Benjamini–Hochberg *p-*value correction, with significant differences indicated by different lower-case letters. RNA samples were isolated from 10 individual plants of each line to assay for each endornavirus (i.e., n = 10 plants per line per endornavirus). Wairimu = Wairimu Dwarf

**Table 8:** The effects of *Phaseolus vulgaris Endornaviruses* 1 and 2 on the accumulation of bean common mosaic necrosis virus (BCMNV) RNA accumulation

Endornavirus Content	Plant Line	Mean relative BCMNV RNA accumulation ± SEM*
Endornavirus-free	GLP24	2.70 ± 0.85^a^
	KATX56	0.67 ± 0.14^b^
	Wairimu	1.55 ± 0.53^ab^
PvEV1	RED40	3.15 ± 1.32^a^
PvEV1 & PvEV2	RWR2245	0.022 ± 0.0029^c^

^*^Reverse transcription-quantitative polymerase chain reaction assays with appropriate primers (Additional file 1: Table S2) were used to measure endornavirus RNA accumulation in twelve cultivars of *Phaseolus vulgaris* (Additional file 1: Table S1), relative to accumulation of two housekeeping gene transcripts, *PvActin 11* and *PvUnknown 1* [29] in plants of the indicated common bean lines infected with BCMNV. There were statistically significant differences (*p* < 0.05) in relative BCMNV RNA steady-state accumulation across the cultivars as determined by Kruskal–Wallis (χ^2^ = 29.275, df = 4, *p =* 0.000006873, n = 50). Pairwise comparisons were performed using a Wilcoxon rank sum test with Benjamini–Hochberg *p-*value correction, with statistically significant differences indicated by different lower-case letters. RNA samples were isolated from 10 individual plants of each line to assay for each endornavirus (i.e., n = 10 plants per line per endornavirus). Wairimu = Wairimu Dwarf

### Seedborne transmission of BCMV and CMV appears to be unaffected by endornaviruses

Seedborne transmission of acute viruses can occur at high frequencies in many common bean varieties [21] and previous work indicated that endornaviruses influence seed production [15]. We investigated if seedborne transmission of two important seed-transmitted acute viruses of common bean, CMV and BCMV, was affected by the presence of endornaviruses using the Wairimu Dwarf line, which contains no endornaviruses, and RED40, which carries PvEV1, and lines RWR2245 (contains PvEV1 and PvEV2), and RWR2075 (contains PvEV1, PvEV2 and PvEV3). Seeds were collected from plants infected with CMV or BCMV, germinated and seedling leaf tissue tested by DAS-ELISA for the presence of these viruses (Table 9; Additional file 3: Spreadsheet S2). The seed transmission data was fitted to binomial regression models. The analysis indicated that the rate of seedborne transmission did not vary significantly between the different common bean lines (*p =* 0.0892).

**Table 9:** The rates of seedborne transmission of the acute viruses bean common mosaic virus (BCMV) and cucumber mosaic virus (CMV) in bean lines with or without endornavirus infection

Common bean Line*	Acute Viral Infection	Number of progeny seedlings tested	Number of progeny testing positive in ELISA	Rate of seedborne transmission (%)
Wairimu Dwarf	CMV	53	0	0.000
	BCMV	57	4	7.018
RED40	CMV	17	1	5.882
	BCMV	43	6	13.953
RWR2245	CMV	39	3	7.692
RWR2075^†^	CMV	20	0	0.000

^*^Endornavirus content: Wairimu Dwarf, no endornaviruses present; RED40, PvEV1; RWR2245, PvEV1 and PvEV2; RWR2075, PvEV1, PvEV2 and PvEV3

^†^RWR2075 seed production occurred at a very low rate for both mock-inoculated and CMV-infected plants

Plants of line RED40 (which carries PvEV1) produced the highest numbers of seeds, compared to the other lines tested, and BCMV and CMV infection caused marked decreases in the number but not the mass of seeds (Table 10; Additional file 3: Spreadsheet S2). Neither BCMV nor CMV caused notable changes in the number or mass of seeds produced by the endornavirus-free line Wairimu Dwarf (Table 10). CMV infection decreased seed number but not seed mass in plants of line RWR2245 (which harbours PvEV1 and PvEV2). Remarkably, CMV infection appeared to increase seed number and mass in RWR2075, which carries PvEV1, PvEV2 and PvEV3, and which in the absence of CMV infection, produced the lowest number of seeds of the lines tested (Table 10). The results imply that the endornavirus complement of a common bean line does not appear to influence in a consistent manner either its seed production, or its susceptibility to the seedborne transmission of acute viruses.

**Table 10:** The effects of cucumber mosaic virus (CMV) and bean common mosaic virus (BCMV) infection on seed yield in bean varieties carrying different complements of *Phaseolus vulgaris* endornaviruses 1, 2 and 3

Common bean line*	Acute viral infection	Mean number of seeds per plant	Mean seed mass (g)
Wairimu Dwarf	Mock	6.93	0.366
	CMV	7.07	0.439
	BCMV	7.92	0.373
RED40	Mock	29.73	0.282
	CMV	1.27	0.233
	BCMV	6.13	0.232
RWR2245	Mock	7.80	0.245
	CMV	4.27	0.251
RWR2075^†^	Mock	0.60	0.031
	CMV	2.00	0.349

^*^Endornavirus content: Wairimu Dwarf, no endornaviruses present; RED40, PvEV1; RWR2245, PvEV1 and PvEV2; RWR2075, PvEV1, PvEV2 and PvEV3

^†^RWR2075 seed production occurred at a very low rate for both mock-inoculated (Mock) and CMV-infected plants

## Discussion

PvEV1 and PvEV3 levels were similar in all lines harbouring these endornaviruses, with PvEV2 being variable in its accumulation. The levels of RNA accumulation for PvEV1, PvEV2 and PvEV3 do not appear to be controlled by interactions with each other. Thus, one of our starting hypotheses, i.e., that synergy or interference occurs between endornaviruses appears not to be correct. This was surprising, since the RNA sequences of these viruses have extensive regions of similarity, which we thought might trigger RNA silencing [32, 33], and the similarity of the viruses also suggested that they might compete for the same host factors. Silencing and competition can act as underlying mechanisms for protection of the host from virus infection by viral cross-protection, sometimes referred to as superinfection exclusion [34, 35]. The most likely conclusion is that in common bean, variation in PvEV2 accumulation is regulated by host factors, but it is puzzling why neither PvEV1 nor PvEV3 levels vary between lines.

Synergism occurs in plants infected by two or more dissimilar viruses, leading to increased titres for at least one of the viruses, especially if one of the viruses possesses a strong RNA silencing suppressor [33]. CMV encodes a strong viral suppressor of RNA silencing, the 2b protein [33], as do the potyviruses BCMV and BCMNV, which encode P1/HCPro proteins [24]. However, in most lines acute viruses induced no statistically significant changes in endornavirus RNA accumulation, although in a few there were decreases and, less frequently, increases. For example, in the line RWR2245 CMV and BCMV induced increases in PvEV1 and PvEV2 accumulation, whilst in BCMNV-infected plants these endornaviruses dropped to a hundredth of their normal levels. MCM2001 was the only line in which CMV induced decreases in the accumulation of all three endornaviruses. Measurement of the accumulation of the RNAs of acute viruses in the presence or absence of endornaviruses also yielded complex results, with no consistent relationship to the presence of the endornaviruses. This suggested that differences in CMV, BCMV and BCMNV titres reflected host properties. We conclude that, in common bean, little or no synergy or interference occurs between endornaviruses and acute viruses.

Plant host developmental stage profoundly influences viral infection cycles [36]. Our investigations of endornavirus-endornavirus and endornavirus-acute virus interactions focused on leaves, but it cannot be ruled out that in other tissues, or at different developmental stages, there would have been more virus-virus interactivity. In most mature plant leaf cells, endornaviruses are unlikely to be replicating actively and so may be less likely to compete with or synergise other viruses. A study of Oryza sativa endornavirus (OsEV) in rice (*Oryza sativa*) showed that this virus accumulated to similar levels (*c*. 100 copies per cell) in various tissues. However, OsEV RNA levels were far higher (*c*. 1000 copies per cell) in actively dividing suspension cell cultures derived from these plants [37]. It appears that endornavirus replication is tied to active cell division. Other work with OsEV indicated that endornavirus RNA is not subject to turnover by RNA-degrading mechanisms. Paradoxically, in rice plants engineered to suppress expression of Dicer-Like 2 (DCL2), an endonuclease component of the antiviral RNA silencing system, OsEV accumulation was diminished or abolished [38]. In rice, DCL2 is expressed at its highest levels in egg cells [39], when endornavirus replication is likely to be active. Thus, it seems that endornaviruses somehow exploit RNA silencing to accumulate.

All the viruses included in this investigation can be seed-transmitted. For the endornaviruses the rate is virtually 100% [2], while BCMV and BCMNV seed transmission rates are in the range 10–30% [40], and for CMV reported rates in common bean are highly variable with some studies detecting < 1% and others up to 100% [25]. Using relatively low numbers of seeds, we found no marked effects of endornaviruses on CMV or BCMV transmission. However, using larger numbers of seeds from a wider range of lines might detect subtle effects. Investigation of the effects of CMV and BCMV infection on seed yield revealed variable outcomes in different bean lines. In Wairimu Dwarf plants, which harbour no endornaviruses, neither CMV nor BCMV engendered marked decreases in seed number or seed mass but, in plants of the line RED40 which harbours PvEV1, both viruses caused decreases in seed number (but not seed mass) and CMV had a similar effect in plants of RWR2245, which harbours PvEV1 and PvEV2. The most curious results were obtained for the line RWR2075, which contains PvEV1, PvEV2, and PvEV3. We noted that plants of RWR2075 yielded comparatively few seeds, but it is possible that growth conditions in the UK are not well suited for this African variety. Interestingly, RWR2075 plants infected with CMV yielded more and heavier seeds. It would be interesting to see if such results, in which CMV appears to be beneficial, occur under this line’s normal cultivation conditions, or if this is related to the stress of growing under glasshouse conditions in the UK. Overall, our investigation found no consistent effects of endornaviruses or acute viruses on seed production.

Our data on the effects of endornaviruses on seed yield contrast with the clear-cut results obtained by Khankhum and Valverde [15]. These workers investigated how various traits differ between plants of two nearly isogenic lines belonging to the Black Turtle Soup common bean variety; one line was endornavirus-free and the other harboured both PvEV1 and PvEV2. Plants of both lines produced similar numbers of pods that contained similar numbers of seeds, however, they found that plants harbouring PvEV1 and PvEV2 produced heavier seeds [15]. The Black Turtle Soup variety comprises a wide variety of North American lineages including heirloom breeds [41]. However, careful breeding of certain Black Turtle Soup lineages has produced sets of nearly isogenic lines. These include plants with and without endornaviruses, and lines with genotypic differences, such as pairs of nearly isogenic lines with the partly dominant *I* locus for BCMV resistance or lacking this locus [42, 43]. We suggest two possible reasons that may explain the contrast between our findings, which suggest that the effects of endornaviruses are variable between common bean lines, and the observations with Black Turtle Soup-derived lines suggesting a beneficial effect of PvEV1 and PvEV2 [15]. Firstly, the benefit of producing larger seeds conferred on plants of Black Turtle Soup by PvEV1 and PvEV2, might be a specific response of this variety to endornaviruses, but may not be conserved in all common bean lineages. Secondly, perhaps the positive effects of endornaviruses on host traits are subtle, and undetectable unless experiments use plant genotypes that are as near identical as possible, as is the case with nearly isogenic lines.

If the second possibility is correct, it may be possible to investigate it further by generating a set of nearly isogenic lines containing various combinations of PvEV1, PvEV2 and PvEV3 for every common bean variety. However, conventional crossing and back-crossing for so many different lines would be time-consuming and impractical. In contrast, by using virus-induced gene silencing it should be feasible to obtain perfectly isogenic lines; for example, by expressing endornavirus-specific RNA silencing sequences using bean pod mottle virus (BPMV) vectors [44]. BPMV would be ideal for producing virus-free seeds since its own seed transmission rate is very low (< 0.1%) [45]. Therefore, a virus-induced gene silencing approach could generate plants and seeds ‘cured’ of endornaviruses to allow phenotypic comparisons between otherwise genetically identical plants with or without endornaviruses present.

## Conclusions

In common bean the endornaviruses PvEV1, PvEV2 and PvEV3 appear not to inhibit or synergize each other, and only PvEV2 RNA showed distinct inter-line variation in its titre. It is likely that differences in accumulation of the acute viruses CMV, BCMV and BCMNV between lines were due to host genotype rather than to the presence of endornaviruses, and there seemed to be no direct synergy or competition between acute viruses and endornaviruses. Endornaviruses appeared to have no consistent effects on seed numbers or seed mass, or seedborne transmission of acute viruses.

### Supplementary Information

**Additional file 1. Table S1** Lines used in this study, with information on their complement of endornaviruses. **Table S2**. Oligonucleotide primer pairs used for reverse transcription-could polymerase chain reactions for the quantification of viral RNA steady-state levels.

**Additional file 2. Spreadsheet S1** Raw Ct data.

**Additional file 3. Spreadsheet S2** Seed weight.

## Data Availability

All relevant data are within the paper and its Additional files. Upon request plant lines will be made available in a timely manner for noncommercial research purposes but lines may be subject to a materials transfer agreement.
