# Supplementary material for: Investigating the interactions of endornaviruses with each other and with other viruses in common bean, Phaseolus vulgaris
Source: Virol J. 2023 Sep 22;20:216. doi: 10.1186/s12985-023-02184-y (PMC10515030; doi:10.1186/s12985-023-02184-y)
Supplement: Supplementary file 1 — Additional file 1. Table S1 Lines used in this study, with information on their complement of endornaviruses. Table S2. Oligonucleotide primer pairs used for reverse transcription-could polymerase chain reactions for the quantification of viral RNA steady-state levels. [file 12985_2023_2184_MOESM1_ESM.docx]

*Supplementary Tables*

**Investigating the interactions of endornaviruses with each other and with other viruses in common bean, *Phaseolus vulgaris***

Thomas J. Brine^1^, Satish Bharathwaj Viswanathan^1^, Alex M. Murphy^1^, Adrienne E. Pate^1^, Francis O. Wamonje^1,2^, and John P. Carr^1^*

1. Department of Plant Sciences, University of Cambridge, Cambridge CB2 3EA, United Kingdom

2. Pest and Pathogen Ecology, National Institute of Agricultural Botany, East Malling ME19 6BJ, United Kingdom

* Communicating author: [jpc1005@cam.ac.uk](mailto:jpc1005@cam.ac.uk)

**SUPPLEMENTARY TABLES**

**Table S1.** Lines used in this study, with information on their complement of endornaviruses.

| **Common Bean Line** | **Endornaviruses Present^†^** | | |
| --- | --- | --- | --- |
|  | **PvEV1** | **PvEV2** | **PvEV3** |
| GLP24 | - | - | - |
| KATX56 | - | - | - |
| WAIRIMU DWARF | - | - | - |
| RED40 | + | - | - |
| RWR1668 | + | - | - |
| GLP1127 | + | - | - |
| KK022 | + | + | - |
| SER16 | + | + | - |
| RWR2245 | + | + | - |
| KK072 | + | + | + |
| RWR2075 | + | + | + |
| MCM2001 | + | + | + |

† The presence (+) or absence (-) of Phaseolus vulgaris endornavirus 1 (PvEV1), PvEV2, or PvEV3[4].

**Table S2** Oligonucleotide primer pairs used for reverse transcription-could polymerase chain reactions for the quantification of viral RNA steady-state levels.

| **Primer Pair ^†^** | **5’ - 3’ Sequence (forward/reverse)** | **Amplicon Length (base pairs)** |
| --- | --- | --- |
| Act11 | TGCATACGTTGGTGATGAGG  AGCCTTGGGGTTAAGAGGAG | 190 |
| Ukn1 | ATTCCCATCATGCAGCAAAG  AGATCCCTCCAGGTCAATCC | 192 |
| PvEV1q1 | \| CACTGACCTTGCCCCTGAAT \| \| --- \| \| GTAATGGCTTGCCATGCGTC \| | 130 |
| PvEV2q1 | \| AGCACAAATGGAACCCGCTA \| \| --- \| \| CGTGGGTGAATGGTGTCTCA \| | 144 |
| PvEV3q1 | \| TGGGCAGTCGTTGAGGAATC \| \| --- \| \| GACAACCGTGGCCTATTGGA \| | 148 |
| CMV_CP | \| GTCTTGTCGCAGCAGCTTTC \| \| --- \| \| ACCCGCGGTCTATTTTTGGT \| | 151 |
| BCMV_CP | \| CGGTACGAGCAAGAGAAGCA \| \| --- \| \| AGGACCCATGCCAAGAAGTG \| | 170 |
| BCMNV_1 | \| AAGGCCCAGCGGATAAAGAC \| \| --- \| \| CTACCGCCAACCATAGGCAA \| | 100 |

† Act11 and Ukn1 are specific for amplification of the *Phaseolus vulgaris Actin 11* and the *PvUnknown 1* transcripts, respectively, and stable housekeeping transcripts identified by Borges et al. [29]. The primer pairs PvEV1q1, PvEV2q1 and PvEV3q1 are targeted to regions within the open reading frames of, respectively, PvEV1, PvEV2 and PvEV3. CMV_CP, BCMV_CP and BCMNV_1 are targeted to regions within the coat protein genes of cucumber mosaic virus, bean common mosaic virus and bean common mosaic necrosis virus, respectively. The annealing temperature for all primer pairs is 60°C.
